# New York

**Published:** 1897-03

**Authors:** 


*New York. Some views and an important decision relating to dehorning.* A decision of great interest to the veterinary medical profession was given by Mr. Justice Davy at a trial term of the Supreme Court, held at Bath, Steuben County, New York, on January 5, 1897. It was in the case of the County of Steuben against Wood. The prosecuting committee of the New York State Veterinary Society caused action to be brought against John Wood, pursuant to Chapter 860, of the Laws of 1895, for the illegal practising of veterinary medicine. The sole and only question involved at the trial was whether the dehorning of cattle was a surgical or non-surgical operation. It was contended on the part of the plaintiff that it was a surgical operation, and that the defendant was guilty of a violation of the statute and was liable to the penalty of fifty dollars for each day's practice. The defence claimed that it was a non-surgical operation, and that any unlicensed persons could perform the operation for hire without violating any statute. The State Veterinary Society employed Lewis Cass, Esq., of Albany, to prosecute the action. It was defended by the best legal talent in Steuben County. Upon the trial of the action Prof. James Law, of the New York State Veterinary College, and Dr. Wm. Henry Kelly, of Albany, Secretary of the State Board of Veterinary Medical Examiners, were called as witnesses for the plaintiff. Both of these eminent gentlemen gave a very lucid and clear description as to what constituted a surgical operation, and, although both were subjected to a very close and rigid cross-examination, the defence failed to shake or in any wise weaken the evidence given on direct examination. At the close of the trial the plaintiff's attorney requested the court to direct verdict for fifty dollars, being one full penalty, which was done. A stay of sixty days was given for the defendant within which to perfect the appeal to the Appellate Division.

This is the first decision under the law of 1895 upon this point. In fact, so far as we have been able to learn, it is the first decision of any court in the United States. This is a great victory for the profession.

Mr. Cass feels confident he will be able to sustain the decision of Judge Davy before the Appellate Division.

